# Salivary Oxytocin Concentration Associates with the Subjective Feeling of Body Ownership during the Rubber Hand Illusion

**DOI:** 10.3389/fnhum.2017.00166

**Published:** 2017-04-07

**Authors:** Masakazu Ide, Makoto Wada

**Affiliations:** ^1^Developmental Disorders Section, Department of Rehabilitation for Brain Functions, Research Institute of National Rehabilitation Center for Persons with DisabilitiesTokorozawa, Japan; ^2^Japan Society for the Promotion of ScienceTokyo, Japan

**Keywords:** rubber hand illusion, salivary oxytocin, body ownership, autistic traits, visuotactile integration, neuroendocrine

## Abstract

Oxytocin is a hormone of the posterior pituitary that promotes lactation, maternal bonding, and birth. Recent studies have shown that oxytocin may modulate social recognition in both sexes, and thus it may be related to empathy. Brain regions that are associated with social recognition and empathy (e.g., the insular cortex) are activated in the rubber hand illusion (RHI), which involves illusory ownership of a rubber hand caused by brush strokes applied synchronously to both a rubber hand and one of the participant's hand, which is hidden from view. It is intriguing to examine whether oxytocin modulates plastic changes in body representation, such as the changes occurring in the RHI. In the present study, we investigated the relationship between salivary oxytocin concentration and the feeling of rubber hand ownership. Brush strokes were applied synchronously or asynchronously to the participant's hand and a rubber hand on different days. Salivary oxytocin was measured before and after the behavioral tasks. We found that participants who had high concentrations of salivary oxytocin tended to feel strong ownership of the rubber hand. We also found that the participants with a high autism spectrum quotient (AQ) score who particularly felt difficulties in social skills and communications tended to feel weak rubber hand ownership. We observed that illusory body ownership was closely linked to social communications and a related neuroendocrine basis. The results of the present study suggest that an individual's salivary oxytocin concentration can predict the extent to which the individual experiences the RHI; furthermore, oxytocin might modulate the sensation of body ownership.

## Introduction

The rubber hand illusion (RHI) describes the feeling of illusory ownership of a rubber hand that arises when brush strokes are applied synchronously to a rubber hand and a participant's hand that is hidden from view, and this feeling is combined with an illusory location of touch (Botvinick and Cohen, 1998; Armel and Ramachandran, 2003; Tsakiris and Haggard, 2005). The illusion results from the integration of vision and touch, which are processed almost simultaneously (e.g., within hundreds of milliseconds of each other; Shimada et al., 2009). Previous imaging studies suggested that the posterior parietal cortex may play an important role in visuotactile integration during the illusion, and the subjective feeling of illusory ownership is related to the activation of the premotor cortex (Ehrsson et al., 2004; Blanke, 2012; Gentile et al., 2013; Limanowski and Blankenburg, 2016). Previous studies reported that the illusion may affect body temperature (Moseley et al., 2008) and the histamine reactivity of the participant's hand (Barnsley et al., 2011). Moreover, Finotti and Costantini (2016) reported that the RHI is strong in patients with autoimmune diseases, such as Coeliac Disease. These results suggest that the effects of this procedure reach beyond simple multisensory integration in the brain. Moreover, previous studies suggested that people with high empathy tend to experience a strong RHI (Asai et al., 2011). Empathy has been reported to be related to the activation of the insular cortex (Bernhardt and Singer, 2012), and damage to the posterior insular cortex results in body ownership disturbances. For example, patients with “anosognosia” typically have a conviction that their limbs function normally, even though the limbs are actually paralyzed. Some of them sometimes feel that their own limbs belong to another person (Karnath et al., 2005). Individuals with autism spectrum disorders (ASDs) who were reported to have disorders of cognitive empathy (Rogers et al., 2007) tend to show smaller proprioceptive drift during the RHI (Paton et al., 2012) compared with neurotypical individuals. Cascio et al. (2012a) reported a delayed occurrence of proprioceptive drift during the RHI, and this effect was prominent during the first block in neurotypical individuals. Moreover, the jerk in a reach-to-grasp movement subsequent to the induction of the RHI was significantly increased in neurotypical individuals. However, this movement was not affected by the RHI in individuals with ASD (Palmer et al., 2015). Although it should be kept in mind that proprioceptive drift is not always accompanied with illusory ownership during the RHI (Holle et al., 2011), previous studies have indicated that disturbances of body representations, which are symptoms of ASD, affect the induction of plastic changes related to body ownership. Several anecdotal reports suggested that individuals with ASD either have an atypical body representation or lack body awareness. Individuals with ASD may show an impaired ability to distinguish between self and others (Russell, 1997), which is a deficit that might be related to disorders of cognitive empathy.

Oxytocin is a hormone of the posterior pituitary that promotes lactation and birth in both rodents and humans. Oxytocin also acts as a neurotransmitter to activate specific neural circuits to motivate parents to nurture, bond with, and protect their offspring (Nagasawa et al., 2012; Stoop, 2012, 2014; Rilling and Young, 2014). Recent studies have revealed that oxytocin modulates social recognition and empathy in both men and women (Kosfeld et al., 2005). Multiple studies have suggested that the effect of oxytocin administration is mediated by modulating social brain networks, including the insular cortex (Riem et al., 2011; Baribeau and Anagnostou, 2015; Wigton et al., 2015), which are related to empathy and seem to be involved in the induction of the RHI. Furthermore, high levels of plasma oxytocin were related to increased activity in brain regions that process social stimuli (Lancaster et al., 2015), and several variants in oxytocin and its receptor genes were found to be associated with ASD (Lopatina et al., 2012). Another line of evidence suggests that oxytocin administration to participants with ASD may be an effective treatment agent for both improving several aspects of social cognition and reducing repetitive behaviors (Cochran et al., 2013). We assume that impairments in sensory motor functions in individuals with ASD underlie disabilities in social cognition, and it is intriguing to examine whether the effect of oxytocin on social cognition is derived from modulation of sensorimotor functions. If this is true, oxytocin might affect plastic changes related to body ownership as well as empathy toward other persons. In addition, it is known that tactile stimuli can increase oxytocin secretion and consequently might facilitate social communications in animals, including humans (McGlone et al., 2014). Synchronous visuotactile stimulation itself might promote the secretion of oxytocin.

Taken together, these results prompted us to further investigate the role of oxytocin in the emergence of illusory ownership during the RHI. In the present study, we tested the following hypotheses. Firstly, oxytocin may enhance plastic changes in body ownership during the RHI through possible modulations in the social brain (e.g., insular cortex), and higher oxytocin levels may be closely associated with illusory ownership. If this is the case, then the oxytocin concentration before induction of the RHI may predict the extent of an individual's subjective sensation during the RHI, and this may be related to the individual's autistic traits. Secondly, oxytocin may increase after the occurrence of the RHI. In this case, plastic changes in body representation resulting from visuotactile integration may promote oxytocin secretion. To test these hypotheses, we analyzed the relationship between the changes in concentrations of salivary oxytocin before and after the RHI task and the sensation of ownership of a rubber hand in neurotypical individuals. We also measured variations in autistic traits of the participants using the autism spectrum quotient (AQ) score (Baron-Cohen et al., 2001; Wakabayashi et al., 2004).

## Materials and methods

### Participants

Fifteen participants [7 female and 8 male participants; mean age of 21.7 ± 0.48 years, mean ± standard error of mean (SEM)] were recruited for this study. All participants had normal or corrected-to-normal (e.g., glasses, contacts) vision and no history of neurological diseases. We scored their handedness (Laterality Quotient, LQ) according to the Edinburgh Inventory (Oldfield, 1971). One participant was ambidextrous (LQ = +30), and 14 participants were right-handed (+50 ≤ LQ ≤ + 100; 88 ± 5.5, mean ± SEM). Additionally, we assessed their autistic traits using the Japanese version of the autism spectrum quotient (AQ) score (Wakabayashi et al., 2004), which was based on the English version (Baron-Cohen et al., 2001). The mean AQ scores were 18.3 ± 1.6 (mean ± SEM). One participant who failed to experience the RHI [due to a score “Sync–Async” (−1.08) falling 2SD (standard deviation) below the average (−0.66)] and who had an AQ score of 38 (i.e., above the criterion for autism spectrum condition) was excluded yielding a total of 15 participants. The study was approved by the ethics committee of the National Rehabilitation Center for Persons with Disabilities. Informed consent was obtained from all participants before the beginning of the experiments.

### Stimulus

**Figure 1:**
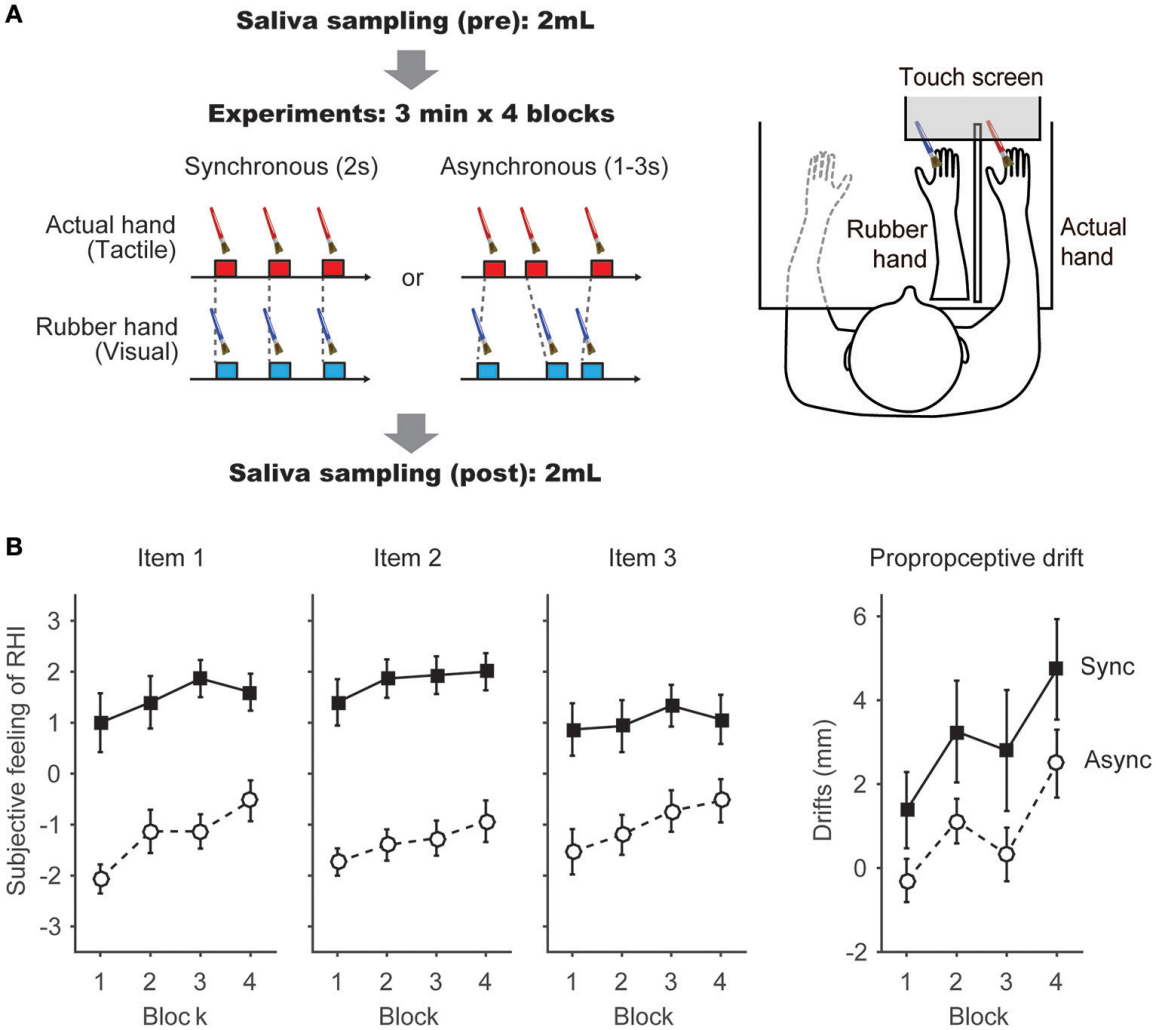
**Apparatus and Task schedules. (A)** Task schedules and apparatus: we adopted two conditions in which the temporal parameters of the visuotactile stimulation were varied. In the Synchronous condition, synchronous brush strokes were applied to the rubber hand and the actual hand at a constant 2-s interval. In the Asynchronous condition, the interval was randomly assigned and ranged between 1 and 3 s. Each condition included 4 blocks. The Synchronous and Asynchronous conditions were conducted on separate days. Before and after the behavioral tasks, 2 mL of saliva were collected from each participant in a centrifuge tube. In each condition, brush strokes were applied to the rubber hand and the participant's hand using a PC-controlled brush. A touch screen was placed above the rubber hand and the participant's hand. Details regarding the apparatus were described in a previous report (Ide and Wada, 2016). **(B)** Subjective feeling of the RHI (Items 1–3) and proprioceptive drift were calculated in each block. Filled squares indicate scores in the Synchronous condition; open circles indicate scores in the Asynchronous condition. Each point indicates the average of each block from 15 participants.

A rubber hand (Otto Bock, Duderstadt, Germany) was placed on the desk in front of the participants (Figure 1A). We used a customized brush-stroking device with two small brushes (Solidray Co Ltd, Japan). We adopted the motor controlled stimulation to avoid any unpredictable influence caused by the experimenter's manual stimulation. The parameters and timings of the stimulation were controlled by a personal computer (VAIO, Sony, Japan) with custom scripts developed in MATLAB (MathWorks, Natick, MA, USA). Stroking with the reciprocating brushes was applied simultaneously to the rubber hand and the participant's right hand with a maximum velocity of 550°/s (80 cm/s). The brush stroking was delivered approximately 90 times in blocks of 3 min. A touch screen (KTMT1921 Add-On Touch Screen for 20–21” LCD & CRT, Keytec, Inc.) was placed obliquely and upward (12 cm above the rubber hand) from the rubber hand and the participant's hand, and it did not occlude the rubber hand from the participant's sight. The participants wore earplugs and noise-canceling headphones to block sounds generated by the DC motors in the device; they also wore a liquid-crystal shutter goggle (TKK2275, Takei Scientific Instruments Co., Ltd) to control their vision. Details of the apparatus were described in our previous report (Ide and Wada, 2016).

### Procedure

A rubber hand was placed in front of the participant; the participant's right hand was placed 13 cm to the right of the rubber hand and hidden from view by an opaque plate. We asked the participant to fixate on the index finger of the rubber hand while the brush stroke was applied. This visuotactile stimulation was applied in two conditions with different temporal parameters (Figure 1A). In the Synchronous condition, the brush strokes were applied synchronously to the rubber hand and the participant's hand at a constant 2-s interval, as described in our previous study (Ide and Wada, 2016). In the Asynchronous condition, the intervals between the brush strokes applied to the rubber hand and actual hand were randomly assigned and varied in a 1–3 s interval. Each condition included 4 blocks, and each block lasted 3 min. The duration of the entire experiment was approximately 20 min, including rest periods between the blocks. The Synchronous and Asynchronous conditions were conducted on separate days (within 4 days) to eliminate the changes in oxytocin concentrations induced by the task itself. The order of the conditions was counterbalanced between the participants. After the completion of the experiment on the first day, the Japanese version of the AQ score (Wakabayashi et al., 2004) was administered to the participants.

In each block, we measured the degree of the RHI. Firstly, the participants were asked to describe the degree of their subjective feeling of the RHI using the questionnaire described in a previous report (Botvinick and Cohen, 1998). The Japanese translated version of the questionnaire (see Ide and Wada, 2016 for reference) was used. The participants described their response on a seven-point magnitude-estimation scale ranging from strongly agree (+3) to strongly disagree (−3). Secondly, we measured the “proprioceptive drift,” which is reported to occur when participants experience the body ownership illusion (Tsakiris and Haggard, 2005). In this phenomenon, participants feel that their own hand is near the rubber hand. The participants were asked to point, with their left hand, the location where they felt their right index finger by using the touch screen and while wearing shutter goggles to limit their vision to the experimental field. We calculated the extent of proprioceptive drift by subtracting the horizontal position the participant pointed to before a block from that observed after the block. Before and after the behavioral tasks, 2 mL of saliva was collected from each participant in a centrifuge tube. These samples were immediately frozen after the experiment and stored in a deep freezer (−80°C). After sample extraction (Szeto et al., 2011), salivary oxytocin was measured using an Oxytocin ELISA kit (Enzo Life Sciences, New York, USA). The concentration ratio of the assay buffer (Oxytocin ELISA kit) was 6, and the detection limit of salivary oxytocin was 2.5 pg/mL. Samples that failed to be detected oxytocin (~0.5 pg/mL) were excluded further analysis (one sample after the Synchronous condition and another one sample before the Asynchronous condition).

### Analysis

According to a previous study (Botvinick and Cohen, 1998), 3 out of 9 items of the questions are related to the RHI [“It seemed as if I were feeling the touch of the paintbrush in the location where I saw the rubber hand touched” (Item 1), “It seemed as though the touch I felt was caused by the paintbrush touching the rubber hand” (Item 2), and “I felt as if the rubber hand were my hand” (Item 3)]. We used a Japanese translated version of these questions as previously reported (Ide and Wada, 2016).

To eliminate the rating bias, we calculated the *Change in subjective feeling of RHI* (“Sync–Async”) by subtracting the individual scores for items 1, 2, and 3 in the Asynchronous condition from the scores in the Synchronous conditions (Ide and Wada, 2016). We also calculated the *Changes in the proprioceptive drift* (“Sync–Async”) by subtracting the extent of proprioceptive drift in the Asynchronous condition from the extent in the Synchronous condition. Statistics were calculated using MATLAB (MathWorks, Natick, MA, USA), R version x64 (http://www.r-project.org/) and G^*^Power3 (Faul et al., 2007).

## Results

In the behavioral task (Figure 1A), brush strokes were applied to the participant's hand and a rubber hand either synchronously or asynchronously (Synchronous and Asynchronous conditions).

### Subjective feeling of RHI

In agreement with previous studies (Botvinick and Cohen, 1998; Armel and Ramachandran, 2003; Tsakiris and Haggard, 2005), most of the participants reported to feel the RHI during the Synchronous condition. We conducted a normality test (Kolmogorov-Smironov test) on the participants' responses to the 9 items and confirmed that these values were normally distributed (>0.15 for all 9 items). Thus, to further investigate the participants' feeling, we performed a 2 × 4 analysis of variance (ANOVA), with two within-subject factors, on the responses to each question, similar to previous studies (Abdulkarim and Ehrsson, 2016; Ide and Wada, 2016). The factors were (i) Condition (Synchronous vs. Asynchronous) and (ii) Block (1st, 2nd, 3rd, and 4th blocks). As for the three questions that were related to the RHI [spatial updating of the body representation (Items 1 and 2) and the subjective feeling of body ownership of the rubber hand (Item 3)] (Botvinick and Cohen, 1998), the analysis revealed a significant main effect of Condition (*F* = 39.6, *p* = 0.00001 < 0.0001, partial η^2^ = 0.74; *F* = 61.12, *p* < 0.00001, partial η^2^ = 0.81; *F* = 60.24, *p* = 0.00001 < 0.0001, partial η^2^ = 0.81) (Figure 1B). We also found a significant main effect of Block in Items 1 and 2 (*F* = 5.78, *p* = 0.002 < 0.01, partial η^2^ = 0.29; *F* = 3.24, *p* = 0.03 < 0.05, partial η^2^ = 0.19). No interactions were observed (*F* = 1.68, *p* = 0.19, partial η^2^ = 0.11; *F* = 0.25, *p* = 0.86, partial η^2^ = 0.02; *F* = 0.82, *p* = 0.49, partial η^2^ = 0.06). The scores of the three questions (Items 1, 2, and 3) in the Synchronous condition were significantly higher than the scores measured in the Asynchronous condition (*T* = 6.29, *p* = 0.00002 < 0.0001, Cohen's *d* = 1.62; *T* = 7.76, *p* = 0.000002 < 0.00001, Cohen's *d* = 2.02; *T* = 7.81, *p* = 0.000002 < 0.00001, Cohen's *d* = 2.08, Figure 1B). Table 1 reports the results of the ANOVAs, including the control questions (Items 4–9).

**Table 1:** **Analysis for the participants' subjective feeling of RHI**.

	**Questions**		***F***	***P***	**Partial *η*^2^**
**1**	**It seemed as if I were feeling the touch of the paintbrush in the location where I saw the rubber hand touched**.	Conditions	39.6	0.00001^***^	0.74
		Blocks	5.78	0.002^**^	0.29
		Conditions × Blocks	1.68	0.19	0.11
**2**	**It seemed as though the touch I felt was caused by the paintbrush touching the rubber hand**.	Conditions	61.12	0.00001^***^	0.81
		Blocks	3.24	0.03^*^	0.19
		Conditions × Blocks	0.25	0.86	0.02
**3**	**I felt as if the rubber hand were my hand**.	Conditions	60.24	0.00001^***^	0.81
		Blocks	2.05	0.12	0.13
		Conditions × Blocks	0.82	0.49	0.06
4	It felt as if my hand were driftinig toward the rubber hand.	Conditions	20.24	0.0005^**^	0.59
		Blocks	2.83	0.05	0.17
		Conditions × Blocks	0.31	0.82	0.02
5	It seemed as if I might have more than one left hand.	Conditions	0.17	0.69	0.01
		Blocks	0.51	0.68	0.04
		Conditions × Blocks	0.93	0.43	0.06
6	It seemed as if the touch I was feeling came from somewhere between my own hand and the rubber hand.	Conditions	0.30	0.59	0.02
		Blocks	2.67	0.06	0.16
		Conditions × Blocks	3.57	0.02^*^	0.20
7	I felt as if my hand were turning rubbery.	Conditions	6.57	0.02^*^	0.32
		Blocks	3.00	0.042^*^	0.18
		Conditions × Blocks	0.45	0.72	0.03
8	It appeared as if the rubber hand were drifting toward my hand.	Conditions	2.42	0.14	0.15
		Blocks	4.25	0.01^*^	0.23
		Conditions × Blocks	0.64	0.59	0.04
9	The rubber hand began to resemble my own hand, in terms of shape, skin tone, freckles or some other visual feature.	Conditions	11.22	0.005^**^	0.44
		Blocks	2.09	0.12	0.13
		Conditions × Blocks	5.23	0.004^**^	0.27

According to a previous study (Botvinick and Cohen, 1998), 3 out of 9 items (Item 1, 2, and 3) are related to the RHI. We used a Japanese translated version of these questions as previously reported (Ide and Wada, 2016). Asterisks indicate the level of statistical significance (

*** *p < 0.001*,

** *p < 0.01*,

* *p < 0.05)*.

### Proprioceptive drift

The analysis revealed a significant effect of Condition (*F* = 5.90, *p* = 0.03 < 0.05, partial η^2^ = 0.30) and Block (*F* = 0.75, *p* = 0.0004 < 0.01, partial η^2^ = 0.35) on the magnitude of proprioceptive drift (Figure 1B). However, the interaction between these factors failed to reach statistical significance (*F* = 0.11, *p* = 0.95, partial η^2^ = 0.008). We found that the proprioceptive drift was significantly larger in the Synchronous condition than in the Asynchronous condition (*T* = 2.43, *p* = 0.03 < 0.05, Cohen's *d* = 0.15). In addition, the proprioceptive drift in the fourth block was significantly larger compared to the drift measured in blocks 1 and 3 (*T* = 4.36, *p* = 0.004 < 0.01, Cohen's *d* = 0.85; *T* = 3.42, *p* = 0.004 < 0.01, Cohen's *d* = 0.53, respectively). This result is in agreement with previous studies (Tsakiris and Haggard, 2005; Ide, 2013; Kalckert and Ehrsson, 2014; Figure 1B). In the subsequent blocks, the proprioceptive drift in the Asynchronous condition showed increased values, especially in the fourth block. After 12 min of asynchronous stroking (i.e., 3 min × 4 blocks), the subjective feelings (Items 1–3) was found to be dissociated from the proprioceptive drift, as previously reported in experiments where intermittent asynchronous stroking was applied (Rohde et al., 2011).

### Relationship between salivary oxytocin and RHI

**Figure 2:**
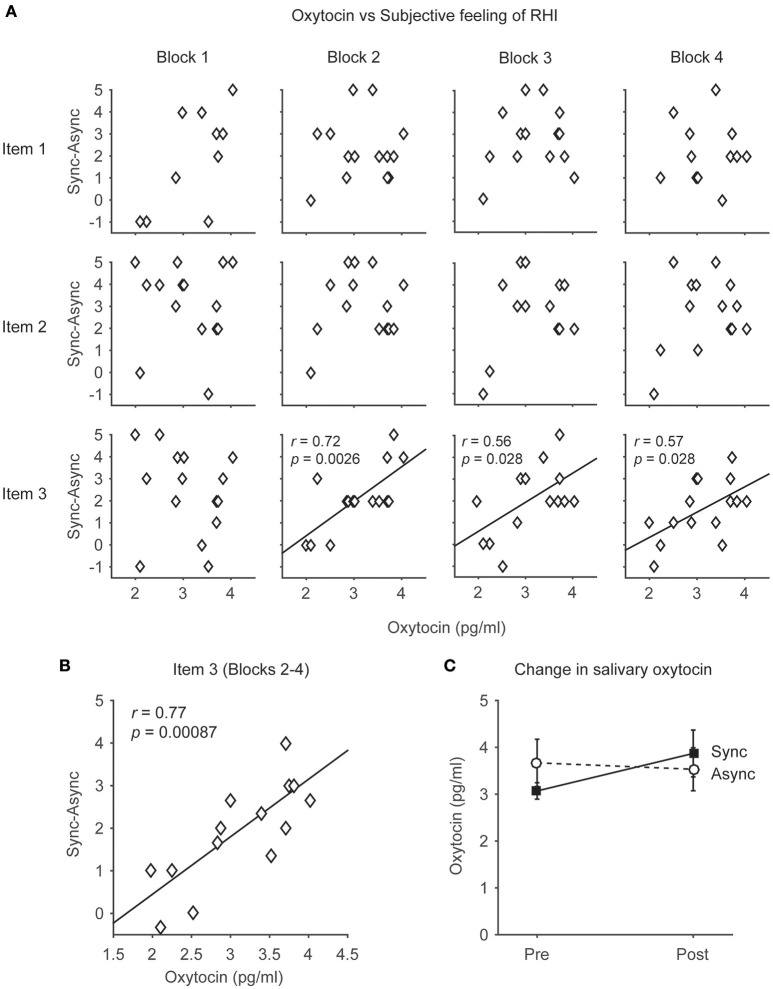
**The relationship between the salivary oxytocin concentration and subjective feeling of the rubber hand illusion (RHI). (A)** Distributions of the salivary oxytocin concentration before the task and subjective feelings of RHI (Items 1, 2, and 3) in each block (1–4). Note that the participants who had higher concentrations of salivary oxytocin tended to feel stronger ownership of the rubber hand in the RHI (Item 3). Each data point indicates the data from the 15 participants. **(B)** Distributions of the salivary oxytocin concentration before the task and subjective feelings of RHI (Items 3) in blocks 2–4. Each data point indicates the averaged data of blocks 2–4 from 15 participants. **(C)** Changes in salivary oxytocin concentration before and after the task (*n* = 14). Note that oxytocin concentrations did not change after the occurrence of the RHI both in the Synchronous and Asynchronous conditions.

In the present study, salivary oxytocin was measured before and after the behavioral tasks (Figure 1A). We compared the relationship between salivary oxytocin concentration (before the behavioral task in the Synchronous condition) and the subjective feeling of the RHI. The change in the illusory feeling attributed to the RHI was calculated by subtracting the response in the Asynchronous condition from the response scored in the Synchronous condition (“Sync–Async”). We found that the salivary oxytocin concentration was positively correlated with a change in the sensation of body ownership of the rubber hand (Item 3, *N* = 15) in Blocks 2–4 (*r* = 0.72, *p* = 0.0026 < 0.01, Power *(1-*β*)* = 0.9; *r* = 0.56, *p* = 0.028 < 0.05, Power *(1-*β*)* = 0.62; *r* = 0.57, *p* = 0.028 < 0.05, Power *(1-*β*)* = 0.64, respectively, Pearson product-moment correlation coefficient, Figure 2A, Table 2). When data from Blocks 2–4 were combined for better signal-to-noise ratio, strong correlation was observed (*r* = −0.77, *p* = 0.00087 < 0.001, Power *(1-*β*)* = 0.96, Figure 2B), and the correlation was still significant with combined data from Blocks 1–4 (*r* = 0.57, *p* = 0.028 < 0.05, Power *(1-*β*)* = 0.64). In addition, the salivary oxytocin concentration was also directly correlated with a score in the Synchronous condition (Item 3) in Blocks 2–4 (*r* = 0.76, *p* = 0.0010 < 0.01, Power *(1-*β*)* = 0.95; *r* = 0.75, *p* = 0.012 < 0.05, Power *(1-*β*)* = 0.94; *r* = 0.62, *p* = 0.014 < 0.05, Power *(1-*β*)* = 0.74, respectively). These results indicated that participants who had high concentrations of salivary oxytocin tended to feel a strong ownership of the rubber hand caused by the RHI. In contrast, no significant correlation between salivary oxytocin concentrations and other questions related to spatial changes in body representation (Items 1 and 2; Figure 2A, Table 2) or proprioceptive drift (Table 2) was observed. These data provide experimental support to our first hypothesis indicating that oxytocin enhanced plastic changes in body ownership during the RHI. In addition, no significant correlation was found between the salivary oxytocin concentration and change in the score of the control questions (Items 4–9; Table 2).

**Table 2:** **Correlations between the salivary oxytocin concentration and extents of RHI in each block**.

**Item numbers**	**Block 1**	**Block 2**	**Block 3**	**Block 4**
**Item 1**	*r* = 0.07	*r* = −0.22	*r* = −0.12	*r* = −0.01
	*p* = 0.82	*p* = 0.43	*p* = 0.66	*p* = 0.97
	Power *(1-β)* = 0.26	Power *(1-β)* = 0.47	Power *(1-β)* = 0.35	Power *(1-β)* = 0.11
**Item 2**	*r* = −0.07	*r* = −0.11	*r* = 0.11	*r* = 0.003
	*p* = 0.80	*p* = 0.69	*p* = 0.71	*p* = 0.99
	Power *(1-β)* = 0.27	Power *(1-β)* = 0.33	Power *(1-β)* = 0.33	Power *(1-β)* = 0.05
**Item 3**	*r* = −0.20	*r* = 0.72	*r* = 0.56	*r* = 0.57
	*p* = 0.48	*p* = 0.0026^**^	*p* = 0.028^*^	*p* = 0.028^*^
	Power *(1-β)* = 0.44	Power *(1-β)* = 0.85	Power *(1-β)* = 0.75	Power *(1-β)* = 0.75
Item 4	*r* = 0.25	*r* = 0.09	*r* = 0.23	*r* = 0.06
	*p* = 0.36	*p* = 0.76	*p* = 0.40	*p* = 0.82
	Power *(1-β)* = 0.50	Power *(1-β)* = 0.30	Power *(1-β)* = 0.48	Power *(1-β)* = 0.25
Item 5	*r* = −0.27	*r* = −0.10	*r* = −0.04	*r* = 0.08
	*p* = 0.33	*p* = 0.71	*p* = 0.87	*p* = 0.78
	Power *(1-β)* = 0.52	Power *(1-β)* = 0.32	Power *(1-β)* = 0.21	Power *(1-β)* = 0.28
Item 6	*r* = 0.03	*r* = −0.08	*r* = 0.13	*r* = 0.18
	*p* = 0.92	*p* = 0.77	*p* = 0.66	*p* = 0.53
	Power *(1-β)* = 0.17	Power *(1-β)* = 0.29	Power *(1-β)* = 0.35	Power *(1-β)* = 0.42
Item 7	*r* = 0.12	*r* = 0.18	*r* = 0.59	*r* = 0.02
	*p* = 0.66	*p* = 0.53	*p* = 0.02	*p* = 0.94
	Power *(1-β)* = 0.35	Power *(1-β)* = 0.42	Power *(1-β)* = 0.77	Power *(1-β)* = 0.14
Item 8	*r* = 0.21	*r* = 0.21	*r* = 0.12	*r* = 0.23
	*p* = 0.45	*p* = 0.45	*p* = 0.67	*p* = 0.40
	Power *(1-β)* = 0.46	Power *(1-β)* = 0.46	Power *(1-β)* = 0.35	Power *(1-β)* = 0.48
Item 9	*r* = −0.08	*r* = 0.44	*r* = 0.13	*r* = 0.10
	*p* = 0.77	*p* = 0.10	*p* = 0.65	*p* = 0.73
	Power *(1-β)* = 0.29	Power *(1-β)* = 0.66	Power *(1-β)* = 0.36	Power *(1-β)* = 0.31
**Proprioceptive drift**	*r* = −0.13	*r* = −0.09	*r* = −0.19	*r* = 0.10
	*p* = 0.65	*p* = 0.76	*p* = 0.50	*p* = 0.72
	Power *(1-β)* = 0.36	Power *(1-β)* = 0.29	Power *(1-β)* = 0.43	Power *(1-β)* = 0.32

Asterisks indicate the level of statistical significance (

** *p < 0.01*,

* *p < 0.05)*.

### Effect of brush stroking on the salivary oxytocin concentration

We investigated the changes in salivary oxytocin concentrations before and after the behavioral task in both the Synchronous and Asynchronous conditions (Figure 2C). In the Synchronous condition, the salivary oxytocin concentrations before and after the RHI task were 3.1 ± 0.18 pg/mL (mean ± SEM) and 3.9 ± 0.52 pg/mL (*n* = 14), respectively. In the Asynchronous condition, the salivary oxytocin concentrations before and after the RHI task were 3.7 ± 0.52 pg/mL (mean ± SEM) and 3.5 ± 0.48 pg/mL (*n* = 14), respectively. We also performed a 2 × 2 ANOVA, with two within-subject factors, on the salivary oxytocin concentration. The main factors were (i) Condition (Synchronous vs. Asynchronous) and (ii) Sampling point (Before vs. After). The analysis revealed no significant main effect of Condition (*F* = 0.08, *p* = 0.78, partial η^2^ = 0.01) and Sampling point (*F* = 0.82, *p* = 0.38, partial η^2^ = 0.06). No significant interactions were found (*F* = 3.41, *p* = 0.09, partial η^2^ = 0.21). These results failed to support our second hypothesis, suggesting that under the present experimental conditions oxytocin concentration does not change after the occurrence of the RHI.

### Relationship between autistic traits and RHI

**Figure 3:**
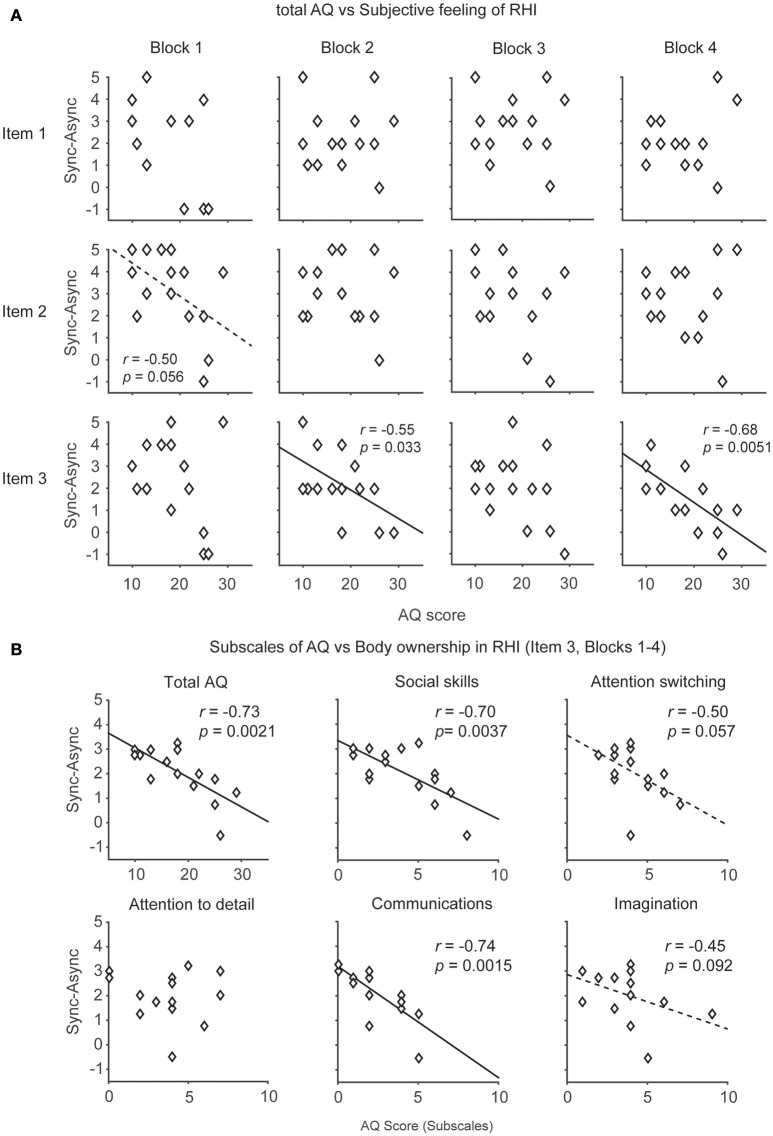
**The relationship between the autism spectrum quotient (AQ) score and subjective feeling of the rubber hand illusion (RHI). (A)** Distributions of the AQ score and subjective feelings of RHI (Items 1, 2, and 3) in each block (1–4). **(B)** Distributions of the AQ score (including subscales) and ownership sensation in the RHI (Item 3). The participants who had difficulties in social skills and communications tended to feel weak ownership sensation in the RHI. Each data point indicates the averaged data of 4 blocks from 15 participants.

We also compared the relationship between the AQ score (Wakabayashi et al., 2004) and the subjective feeling driven by the RHI because recent evidence showed that individuals with ASD have limited subjective responses in the RHI (Paton et al., 2012). The analysis of Item 3 (body ownership of the rubber hand) revealed that the AQ score was negatively correlated with illusory ownership in Blocks 2 and 4 (*r* = −0.55, *p* = 0.033 < 0.05, Power *(1-*β*)* = 0.6; *r* = −0.68, *p* = 0.0051 < 0.01, Power *(1-*β*)* = 0.85, respectively, Pearson product-moment correlation coefficients, Figure 3A, Table 3). When data from Blocks 1–4 were combined for better S/N ratio, strong correlation was observed (*r* = −0.73, *p* = 0.0021 < 0.01, Power *(1-*β*)* = 0.92, Figure 3B). Furthermore, two of the subscales of the AQ score (difficulties in “Social skills” and “Communications”) showed a strong negative correlation with illusory ownership (*r* = −0.70, *p* = 0.0037 < 0.01, Power *(1-*β*)* = 0.88; *r* = −0.74, *p* = 0.0015 < 0.01, Power *(1-*β*)* = 0.93, respectively, Pearson product-moment correlation coefficient, Figure 3B). Two other subscales (difficulties in “Attention switching” and “Imagination”) showed a similar, although not significant, trend (*r* = −0.50, *p* = 0.057, Power *(1-*β*)* = 0.5; *r* = −0.45, *p* = 0.092, Power *(1-*β*)* = 0.41). The other subscale showed no significant correlation [“Attention to detail,” *r* = −0.014, *p* = 0.96, Power *(1-*β*)* = 0.05]. These results suggest that the participants with high AQ scores, especially those who felt difficulties in social communications, tended to feel a weak illusory ownership sensation. Furthermore, we found that the difficulties in “Communications” in AQ score was negatively correlated with the salivary oxytocin concentration (*r* = −0.55, *p* = 0.034 < 0.05, Power *(1-*β*)* = 0.6). In contrast, the total AQ and the other subscales were failed to reach statistical significance [Total AQ, “Social skills,” “Attention switching,” “Attention to detail,” “Imagination,” *r* = −0.38, *p* = 0.17, Power *(1-*β*)* = 0.29; *r* = −0.23, *p* = 0.42, Power *(1-*β*)* = 0.13; *r* = 0.080, *p* = 0.78, Power *(1-*β*)* = 0.06; *r* = −0.078, *p* = 0.78, Power *(1-*β*)* = 0.06; *r* = −0.40, *p* = 0.14, Power *(1-*β*)* = 0.32, respectively]. These results indicate that body ownership as well as communication skills might be modulated by oxytocin.

**Table 3:** **Correlations between the AQ score and extents of RHI in each block**.

**Item numbers**	**Block 1**	**Block 2**	**Block 3**	**Block 4**
**Item 1**	*r* = −0.17	*r* = −0.005	*r* = −0.01	*r* = −0.06
	*p* = 0.53	*p* = 0.99	*p* = 0.96	*p* = 0.82
	Power *(1-β)* = 0.42	Power *(1-β)* = 0.07	Power *(1-β)* = 0.12	Power *(1-β)* = 0.25
**Item 2**	*r* = −0.50	*r* = −0.12	*r* = −0.17	*r* = −0.02
	*p* = 0.06	*p* = 0.68	*p* = 0.54	*p* = 0.93
	Power *(1-β)* = 0.71	Power *(1-β)* = 0.34	Power *(1-β)* = 0.42	Power *(1-β)* = 0.15
**Item 3**	*r* = −0.32	*r* = −0.55	*r* = −0.39	*r* = −0.68
	*p* = 0.24	*p* = 0.03^*^	*p* = 0.15	*p* = 0.005^**^
	Power *(1-β)* = 0.57	Power *(1-β)* = 0.74	Power *(1-β)* = 0.62	Power *(1-β)* = 0.83
Item 4	*r* = −0.50	*r* = −0.31	*r* = −0.52	*r* = −0.35
	*p* = 0.06	*p* = 0.27	*p* = 0.04^*^	*p* = 0.21
	Power *(1-β)* = 0.71	Power *(1-β)* = 0.55	Power *(1-β)* = 0.72	Power *(1-β)* = 0.59
Item 5	*r* = 0.06	*r* = −0.11	*r* = −0.06	*r* = −0.14
	*p* = 0.84	*p* = 0.70	*p* = 0.83	*p* = 0.62
	Power *(1-β)* = 0.24	Power *(1-β)* = 0.33	Power *(1-β)* = 0.25	Power *(1-β)* = 0.38
Item 6	*r* = −0.01	*r* = −0.27	*r* = −0.14	*r* = −0.23
	*p* = 0.98	*p* = 0.34	*p* = 0.62	*p* = 0.42
	Power *(1-β)* = 0.08	Power *(1-β)* = 0.52	Power *(1-β)* = 0.37	Power *(1-β)* = 0.47
Item 7	*r* = −0.18	*r* = −0.10	*r* = −0.45	*r* = 0.04
	*p* = 0.51	*p* = 0.72	*p* = 0.09	*p* = 0.88
	Power *(1-β)* = 0.43	Power *(1-β)* = 0.32	Power *(1-β)* = 0.67	Power *(1-β)* = 0.21
Item 8	*r* = −0.42	*r* = −0.03	*r* = −0.15	*r* = −0.02
	*p* = 0.12	*p* = 0.91	*p* = 0.59	*p* = 0.96
	Power *(1-β)* = 0.65	Power *(1-β)* = 0.17	Power *(1-β)* = 0.39	Power *(1-β)* = 0.12
Item 9	*r* = −0.19	*r* = −0.56	*r* = −0.46	*r* = −0.25
	*p* = 0.50	*p* = 0.03^*^	*p* = 0.07	*p* = 0.36
	Power *(1-β)* = 0.45	Power *(1-β)* = 0.75	Power *(1-β)* = 0.68	Power *(1-β)* = 0.50
**Proprioceptive drift**	*r* = −0.23	*r* = −0.19	*r* = −0.45	*r* = −0.31
	*p* = 0.42	*p* = 0.50	*p* = 0.09	*p* = 0.27
	Power *(1-β)* = 0.47	Power *(1-β)* = 0.44	Power *(1-β)* = 0.67	Power *(1-β)* = 0.55

Asterisks indicate the level of statistical significance (

** *p < 0.01*,

* *p < 0.05)*.

In addition, no correlation was observed between the AQ score and any of the questions related to spatial changes in body representation (Items 1 and 2, Figure 3A, Table 3) or proprioceptive drift (Table 3).

## Discussion

The present results indicate that individual salivary oxytocin concentrations are associated with variation in the individual experiences during the RHI. Although the sample size of the present experiment was relatively small, the correlations observed in the present results have important suggestions that neuroendocrine states may be associated with plastic changes in body ownership (Item 3). In line with our first hypothesis, oxytocin might modulate the feeling of body ownership.

Previous studies have suggested that neural activity in the premotor cortex is correlated with experienced subjective ownership; in contrast, activity in the posterior parietal cortex may be related to multisensory integration occurring during the RHI task (Ehrsson et al., 2004, 2005). Interestingly, oxytocin acts as a neuromodulator in the cerebral cortex (Stoop, 2012). Oxytocin administration in children with ASD enhances activity of their premotor cortex during the execution of a social judgment task (Gordon et al., 2013). We speculate that oxytocin also modulates activity in the premotor cortex and its connected regions when participants experience illusory body ownership. These speculations are supported by the present results, indicating that the salivary oxytocin concentration is positively correlated with the subjective feeling of body ownership but not with spatial updating of the body representation (Items 1, 2, and proprioceptive drift). Moreover, the insular cortex and anterior cingulate gyrus are thought to be plausible targets of oxytocin's modulatory effects because both these regions are related to body representation and self-consciousness (Tsakiris et al., 2007; Blanke, 2012). The insular cortex is known to be critically involved in empathy, and its activity is modulated by oxytocin (Rilling and Young, 2014). We, therefore, speculate that oxytocin increases the saliency of touch inputs by modulating the activity of the social brain network, including the insular cortex; this modulation may enhance the RHI by allocating more resources to touch sensation than proprioception. We also found that participants with high autistic traits tended to feel weak body ownership in the RHI. The result was partly consistent with previous studies reporting that individuals with ASD have disturbances in body representations (Cascio et al., 2012a; Paton et al., 2012; Palmer et al., 2015). Previous studies investigating motor learning (Haswell et al., 2009) and tactile temporal order judgment (Wada et al., 2014) suggested that the disturbances of body representations reported in individuals with ASD are explainable by a strong weight on proprioception. Interestingly, in the present study, we found that a subjective feeling of difficulty in social skills and communications (subscales of the AQ score) was strongly correlated with weak body ownership of the rubber hand. These results indicate that disturbances in body representations might be linked to deficits in social communications in individuals with high autistic traits. These results are interesting because illusory body ownership and social communication seem to be different entities. However, several studies (Tsakiris et al., 2007; Asai et al., 2011), including the present study, suggested that the illusory body ownership is closely linked to empathy and the related neuroendocrine basis. Thus, part of the RHI might be affected by neural activities related to empathy. A bold suggestion is that empathy might be partly derived from a type of extension of body representation caused by synchronous sensory inputs such as the RHI. Our current findings add a new perspective on the relationship between body ownership and the social brain network.

In contrast to the pattern of results observed for the illusory body ownership of the rubber hand, neither the spatial changes in body representation (Items 1 and 2) nor proprioceptive drift were correlated with the concentration of salivary oxytocin or the AQ score. Oxytocin may not have a modulatory effect on the spatial changes in body representation, which seems to be directly induced by multisensory integration. In addition, we previously demonstrated that the rubber hand presentation promotes visuotactile interactions during a tactile temporal order judgment task, particularly in individuals with high autistic traits (Wada and Ide, 2016). From these results, we speculate that there are different mechanisms for spatial updating and plastic changes in body ownership. In line with the speculation, proprioceptive drift arises even when participants do not feel ownership of the rubber hand (Holle et al., 2011; Rohde et al., 2011; Lloyd et al., 2013), although a subjective feeling of the illusion causes proprioceptive drift (Abdulkarim and Ehrsson, 2016). This suggests that oxytocin may modulate plastic changes in body ownership after visuotactile integration rather than during the visuotactile integration itself.

Our subsequent analysis failed to provide experimental support for our second hypothesis, which was that oxytocin concentration may increase after induction of the RHI. This suggests that plastic changes in body representation induced by visuotactile integration may not be directly related to the secretion of oxytocin. However, recent studies suggested that tactile stimuli increase oxytocin secretion (Okabe et al., 2015), and this stimulation might facilitate social communications in animals, including humans (McGlone et al., 2014). Another study showed that these effects are induced by brush stroking applied at 1–10 cm/s, which elicited an “affective touch” via C-tactile (CT) afferents (Löken et al., 2009). Furthermore, the “affective touch” promotes an enhancement of the subjective feeling of ownership in the RHI (Crucianelli et al., 2013; Lloyd et al., 2013; van Stralen et al., 2014). It also induces neural activation in the insular cortex (Cascio et al., 2012b), which is a target of oxytocin administration (Riem et al., 2011). Further experiments are required to determine whether oxytocin concentration increases after the RHI when affective tactile stimulation is delivered. Studies to examine whether the increase effectively enhances the feeling of illusory body ownership through positive feedback are also required. In future studies, it will be intriguing to evaluate the changes in empathy in each participant in addition to the autistic traits, because oxytocin receptor genetic variation is also known to be related to empathy profiles of each person (Rodrigues et al., 2009).

Oxytocin may also modulate other aspects of the RHI and act both as a neuromodulator and a hormone. Ehrsson et al. (2007) reported that threatening the rubber hand during the RHI produces a pattern of regional brain activation in the insular and anterior cingulate cortices (i.e., brain regions responsible for the processing of anxious stimuli and interoceptive awareness) that mimics the pattern of activity evoked by a threat applied to the real hand. Recent evidence has shown that oxytocin enhances the effects of emotional context on the subjective unpleasantness of experimental heat pain (Zunhammer et al., 2016). In addition, oxytocin has been shown to exert a neuromodulatory effect on social brain networks (Riem et al., 2011; Baribeau and Anagnostou, 2015; Wigton et al., 2015). Thus, oxytocin might enhance threat-evoked responses during the RHI, although oxytocin is also known to have an analgesic effect on acute pain (Rash et al., 2014). Moreover, the RHI decreases the temperature (Moseley et al., 2008) and enhances the histamine reactivity (Barnsley et al., 2011) of the participant's hand. In addition, oxytocin exerts a modulatory effect on the hypothalamus, an important hub of the autonomic nervous system. However, the specific effects of the body ownership illusion on temperature (Moseley et al., 2008) and histamine reactivity (Barnsley et al., 2011) only on the arm is unlikely to occur in this case. This is because oxytocin works as a neuromodulator and hormone, which should have an effect on the whole body state (Stoop, 2012). Understanding the contribution of oxytocin to these effects is an interesting topic that needs further research.

Our results suggest that oxytocin might promote plastic changes in body representation. According to our results, the modulatory effects would be mainly evident on the subjective experience of body ownership, rather than on spatial changes in body representation. In contrast, previous studies have shown that individuals with ASD have a slower emergence of the RHI in comparison to non-ASD control participants (Cascio et al., 2012a). This might be due to an excessive reliance on proprioceptive (Haswell et al., 2009) and interoceptive (Schauder et al., 2015) inputs in individuals with ASD. Recent studies have reported that inhalation of oxytocin might be effective in both enhancing social cognition and improving deficits in sensorimotor disorders related to body representations (Andari et al., 2010; Watanabe et al., 2014). Taken together, the present study suggests that inhalation of oxytocin might also improve sensorimotor disorders related to body representation in individuals with ASD.

## Author contributions

MI and MW conducted the experiments, and analyzed the data; MW and MI designed the experiments and wrote the paper.

### Conflict of interest statement

The authors declare that the research was conducted in the absence of any commercial or financial relationships that could be construed as a potential conflict of interest.
